# Mechanisms Underlying the Antinociceptive, Antiedematogenic, and Anti-Inflammatory Activity of the Main Flavonoid from *Kalanchoe pinnata*

**DOI:** 10.1155/2014/429256

**Published:** 2014-12-11

**Authors:** Raquel Teixeira Ferreira, Marcela Araújo Soares Coutinho, David do Carmo Malvar, Elson Alves Costa, Iziara Ferreira Florentino, Sônia Soares Costa, Frederico Argollo Vanderlinde

**Affiliations:** ^1^Laboratory of Pharmacology, Department of Physiological Sciences, Institute of Biology, Federal Rural University of Rio de Janeiro, BR 465, Km 07, 23890-000 Seropédica, RJ, Brazil; ^2^Núcleo de Pesquisa de Produtos Naturais, Universidade Federal do Rio de Janeiro, Avenida Carlos Chagas Filho 373, 21941-902 Cidade Universitária, RJ, Brazil; ^3^Universidade Federal de Goiás, Instituto de Ciências Biológicas, Departamento de Ciências Fisiológicas, 74001-970 Goiânia, GO, Brazil

## Abstract

*Kalanchoe pinnata* (KP) is popularly used for treating inflammatory diseases. This study investigated the antinociceptive, antiedematogenic, and anti-inflammatory potential of the subcutaneous administration of KP flower aqueous extract (KPFE), its ethyl acetate (EtOAcF) and butanol (BuOHF) fractions, and the main KP flavonoid [quercetin 3-*O*-*α*-L-arabinopyranosyl (1 → 2) *α*-L-rhamnopyranoside] (KPFV) in mice, as well as its possible mechanisms of action. KPFE (30–300 mg/kg) and KPFV (1–10 mg/kg) inhibited the acetic acid-induced writhing (ID_50_ = 164.8 and 9.4 mg/kg, resp.). KPFE (300 mg/kg), EtOAcF (12 mg/kg), BuOHF (15 mg/kg), or KPFV (0.3–3.0 mg/kg) reduced leukocyte migration on carrageenan-induced pleurisy (ID_50_ = 2.0 mg/kg for KPFV). KPFE (3–30 mg/kg) and KPFV (0.3–3.0 mg/kg) reduced the croton oil-induced ear edema (ID_50_ = 4.3 and 0.76 mg/kg, resp.). KPFE and KPFV reduced the TNF-*α* concentration in the pleural exudates on carrageenan-induced pleurisy test. Moreover, KPFV inhibited COX-1 (IC_50_ = 22.1 *μ*g/mL) and COX-2 (IC_50_ > 50 *μ*g/mL). The selectivity index (COX-1_IC_50__/COX-2_IC_50__) was <0.44. These results indicate that KPFE and KPFV produced antinociceptive, antiedematogenic, and anti-inflammatory activities through COX inhibition and TNF-*α* reduction, revealing that the main flavonoid in KP flowers and leaves plays an important role in the ethnomedicinal use of the plant.

## 1. Introduction

*Kalanchoe pinnata* (Lamarck) Persoon (=* Bryophyllum pinnatum*), one of the most important medicinal species of the family Crassulaceae, is used in folk medicine to treatment of many diseases such as cardiovascular dysfunctions [1] and diabetes [2] and for healing wounds and treating inflammations [3, 4]. The leaves from this plant are the part most commonly used and therefore have been the focus of a variety of chemical and pharmacological studies. Several studies have reported antiviral, antimicrobial, antiulcer, antileishmanial, hepatoprotective, antioxidant, and antihyperglycemic activities from leaves of* K. pinnata* [1, 2, 5]; to review see [4]. Recent studies have shown the potential activity of KP leaves in inhibiting inflammatory events related to allergic airway diseases [6]. Anti-inflammatory activity has also been reported for KP leaves [7–9]. Polyphenolic compounds present in the extract are speculated to be responsible for this activity. Although many studies have been carried out with KP leaves, there are few works devoted to the flowers of this species [3].

Recently we demonstrated the immunosuppressive potential of the aqueous extract from KP flowers. The KPFE proved to be more active than the aqueous leaf extract in inhibiting murine T-cell mitogenesis* in vitro*. In addition, five quercetin-derivative flavonoids isolated from the flower extract showed potent inhibitory activity against murine T-cell mitogenesis as well as on IL-2 and IL-4 production, without cell toxicity [10]. Flavonoids are the main phenolic compounds in the* Kalanchoe* species [3] and are of great pharmacological importance [11]. Some studies have demonstrated a direct relation between the anti-inflammatory effects of various flavonoids and cyclooxygenases 1 and 2 [12], leading to a reduction of important anti-inflammatory mediators such as prostaglandin E_2_ [13]. Additionally, other studies have revealed the efficacy of flavonoids in the reduction of TNF-*α* levels [14–16] through the ability to reduce the expression of some genes linked with proinflammatory events [14] and the circulating levels of TNF-*α* in humans [16].

Considering the promising preliminary results with KP flowers and the relevant therapeutic potential of this species, the aim of this study was to investigate the antinociceptive, antiedematogenic, and anti-inflammatory activities of the KP flowers aqueous extract, the two enriched-flavonoid fractions, and the main KPF flavonoid [quercetin 3-*O*-*α*-L-arabinopyranosyl (1 → 2) *α*-L-rhamnopyranoside] (here denominated as KPFV) on pharmacological inflammation models.

## 2. Material and Methods

### 2.1. Plant Material

Flowers of* Kalanchoe pinnata* (Lamarck) Persoon were collected from specimens cultivated in the UFRJ campus, Brazil, in September 2011, and identified by the botanist M. F. Freitas at the herbarium of the Rio de Janeiro Botanical Garden. A voucher specimen (292.697) is deposited at the Herbarium of the Rio de Janeiro Botanical Garden (Brazil).

### 2.2. Extraction and Isolation

Fresh flowers (2.52 kg) were ground and extracted with distilled water at 20% w/v for 30 min at 50°C. The yield of lyophilized flower extract (KPFE) was 3.82% (90.2 g) from the initial fresh material. Dried flower extract was resuspended in distilled water (2.1 L) and precipitated with EtOH (1 : 1). The soluble fraction (76.9 g) was partitioned with ethyl acetate (1 × 550 mL; 2 × 225 mL), affording EtOAcF (5.58 g; 6.2%, wt/wt of dried KPFE extract). The residual soluble fraction (71.38 g) was then partitioned with *n*-butanol (1 × 550 mL; 2 × 225 mL), affording BuOHF (12.35 g; 13.7%, wt/wt of dried KPFE extract). The flavonoid quercetin 3-*O*-*α*-L-arabinopyranosyl (1 → 2) *α*-L-rhamnopyranoside (KPFV) was isolated (95% purity; retention time 31.2 minutes) according to procedures previously reported [10].

HPLC chromatograms of* Kalanchoe pinnata* flowers extract, organic fractions, and isolated flavonoid [quercetin 3-*O*-*α*-L-arabinopyranosyl (1 → 2) *α*-L-rhamnopyranoside] (KPFV), along with ^1^H and ^13^C-NMR spectra of the flavonoid, are available online in Supplementary Material (at http://dx.doi.org/10.1155/2014/429256).

### 2.3. Drugs

The drugs used were dexamethasone (Decadron, Aché Lab. Farm, São Paulo, Brazil), carrageenan, indomethacin, croton oil (Sigma Chemical Co., St. Louis, USA), and acetone (Merck AG, Darmstadt, Germany), acetic acid, and PBS solution. The lyophilized KP flower extract (KPFE), the butanol (BuOHF) and ethyl acetate (EtOAcF) KP fractions, the flavonoid [quercetin 3-*O*-*α*-L-arabinopyranosyl (1 → 2) *α*-L-rhamnopyranoside] (KPFV), and the drugs were diluted in saline 0.9% in such a concentration so as to allow the administration of 10 mL/kg, by subcutaneous (s.c.) route, for each dose employed. EtOAcF, BuOHF, and KPFV doses were determined according to their yields from KPFE.

### 2.4. Animals

The experimental protocols in the pharmacological assays using adult male Swiss mice (25–35 g) were approved by the local Animal Care and Use Committee (3403/2011/COMEP/UFRRJ).

### 2.5. Acetic Acid-Induced Abdominal Writhing

Groups of mice (*n* = 5-6) were treated subcutaneously with saline, KPFE (30, 100, and 300 mg/kg), KPFV (1, 3, and 10 mg/kg), or the positive control indomethacin (10 mg/kg) 30 min before acetic acid injection (1.2%, 0.1 mL/10 g) and the number of writhings was counted for the following 30 min [17]. The results were expressed as means ± SEM of number of writhings allowing the ID_50_ value calculation.

### 2.6. Carrageenan-Induced Pleurisy

Groups of mice (*n* = 8) were treated subcutaneously with saline, KPFE (300 mg/kg), KP fractions (BuOHF = 15 mg/kg; EtOAcF = 12 mg/kg), KPFV (0.3, 1.0, and 3.0 mg/kg), or dexamethasone (2 mg/kg) 30 min prior to an injection of carrageenan (1% in saline) into the pleural cavity. After four hours the pleural exudate was collected with 1 mL of heparinized PBS and the total number of leukocytes was counted in a Neubauer chamber [18].

### 2.7. Croton Oil-Induced Mice Ear Edema

Groups of mice (*n* = 8–11) were treated subcutaneously with saline, KPFE (3, 10, and 30 mg/kg), KPFV (0.3, 1.0, and 3.0 mg/kg), or dexamethasone (2 mg/kg) 30 min before application of croton oil (2.5% in acetone, 20 *μ*L) on the inner surface of the right ear. The weight difference (Δ) between the right ears (croton oil, 2.5%) and left ears (acetone) was measured four hours after croton oil application [18].

### 2.8. Evaluation of Action Mechanisms

#### 2.8.1. TNF-*α* *Ex Vivo* Measurement

Groups of mice (*n* = 8) were treated subcutaneously with saline, KPFE (300 mg/kg), KPFV (3 mg/kg), or dexamethasone (2 mg/kg) 30 min prior to carrageenan injection (1% in saline; 500 *μ*L/mouse) into the pleural cavity. Four hours after carrageenan administration, the pleural exudate was collected with 1 mL of heparinized PBS. An aliquot was used to evaluate TNF-*α* concentrations in pleural exudate through an immunosorbent assay kit (ELISA) (Ebioscience, San Diego, CA, USA). Results were expressed as means ± SEM of TNF-*α* concentration (pg/mL) [19].

#### 2.8.2. *In Vitro* Cyclooxygenase (COX) Inhibition Assay

The inhibitory effect of KPFV and the positive control indomethacin on COX-1/COX-2 enzymatic activity were determined using a colorimetric COX (ovine) inhibitor screening assay kit (Cayman Chemical, Catalogue number 760111) according to the protocol recommended by the supplier [19]. The range of KPFV concentrations used for evaluation of both COX activities was from 3.125 to 50 *μ*g/mL, while the ranges of indomethacin concentration were from 2.5 to 80 *μ*g/mL and from 18.75 to 600 *μ*g/mL for COX-1 and COX-2 activity, respectively.

### 2.9. Statistical Analysis

Data were statistically analyzed with* GraphPad Prism 5*, and the results were expressed as mean ± SEM. Differences among the groups were calculated using one-way ANOVA followed by* Tukey-Kramer test*, and test data were considered different at a significance level of *P* < 0.05.

## 3. Results

Purification of aqueous extract from KP flowers (KPFE) afforded two enriched-flavonoid fractions: EtOAcF (6.2%) and BuOHF (13.7%).

**Figure 1:**
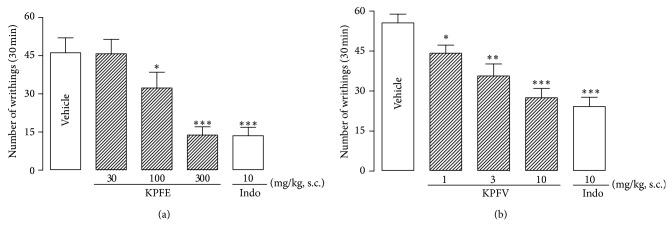
Effect of KPFE (a) and KPFV (b) on the acetic acid-induced abdominal writhing test. KPFE, KPFV, or indomethacin (Indo) was subcutaneously administrated 30 min before acetic acid injection (1.2%, 0.1 mL/10 g). The number of acetic acid-induced abdominal writhings was counted for the following 30 min. ^*^ *P* < 0.05, ^**^ *P* < 0.01, and ^***^ *P* < 0.001 significantly different from the vehicle-treated group. Values represent the mean ± SEM of 5-6 mice.

Pretreatment (s.c.) of mice with KPFE at 100 and 300 mg/kg produced antinociception evidenced by the reduction of the number of acetic acid-induced writhings (w) by 30.1% (32.3 ± 6.2 w) and 70.1% (13.8 ± 3.2 w), respectively (ID_50_ 164.8 mg/kg), comparatively with vehicle group (46.2 ± 5.8 w), while the positive control indomethacin (10 mg/kg) reduced by 70.8% (13.5 ± 3.4 w) and KPFE (30 mg/kg) was ineffective (Figure 1(a)).

The main flavonoid KPFV (1, 3, and 10 mg/kg) also produced a dose-related inhibition of the number of acetic acid-induced writhing by 20.5% (44.2 ± 3.1 w), 35.8% (35.7 ± 4.5 w), and 50.5% (27.5 ± 3.5 w), respectively (ID_50_ 9.4 mg/kg), when compared with vehicle group (55.6 ± 3.3 w). As expected, the positive control indomethacin (10 mg/kg) reduced the number of writhings by 56.5% (24.2 ± 3.5 w) (Figure 1(b)).

**Figure 2:**
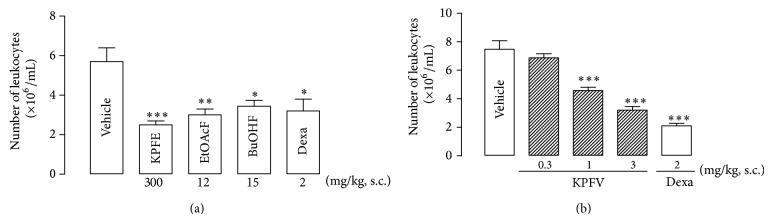
Effect of KPFE, KP fractions (a) and KPFV (b) in the carrageenan-induced pleurisy. KPFE, KP fractions (EtOAcF and BuOHF), KPFV, or dexamethasone (Dexa) was subcutaneously administrated 30 min before carrageenan injection (1%, 0.1 mL/10 g). After four hours, the pleural exudate was collected and the total number of leukocytes was counted in a Neubauer chamber. ^*^ *P* < 0.05, ^**^ *P* < 0.01, and ^***^ *P* < 0.001 significantly different from the vehicle-treated group. Values represent the mean ± SEM of 8 mice.

In the carrageenan-induced pleurisy assay the pretreatment (s.c.) with KPFE (300 mg/kg), EtOAcF (12 mg/kg), BuOHF (15 mg/kg), or dexamethasone (positive control group—2 mg/kg) reduced the leukocyte migration into the pleural cavity by 56.1% (2.5 ± 0.2 leukocytes × 10^6^/mL), 47.3% (3.0 ± 0.3 leukocytes × 10^6^/mL), 39.6% (3.4 ± 0.3 leukocytes × 10^6^/mL), and 43.9% (3.2 ± 0.6 leukocytes × 10^6^/mL), respectively, when compared to the vehicle-treated group (5.7 ± 0.7 leukocytes × 10^6^/mL) (Figure 2(a)).

KPFV (0.3, 1.0, and 3.0 mg/kg) also exhibited a dose-related reduction of leukocyte migration by 8.0% (6.9 ± 0.6 leukocytes × 10^6^/mL), 38.8% (4.6 ± 0.2 leukocytes × 10^6^/mL), and 57.2% (3.2 ± 0.3 leukocytes × 10^6^/mL), respectively (ID_50_ 2.0 mg/kg), whereas the treated with dexamethasone (2 mg/kg), positive control group, inhibited by 71.9% (2.1 ± 0.2 leukocytes × 10^6^/mL) when compared with the vehicle-treated group (7.5 ± 0.6 leukocytes × 10^6^/mL) (Figure 2(b)).

**Figure 3:**
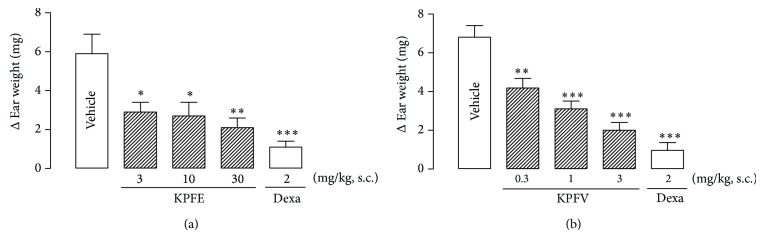
Effect of KPFE (a) and KPFV (b) on the croton oil-induced mice ear edema. KPFE, KPFV, or dexamethasone (Dexa) was subcutaneously administrated 30 min before application of croton oil (2.5% in acetone, 20 *μ*L) or acetone on the inner surface of the right and left ear, respectively. After four hours, the weight difference (Δ) between the right and left ears was measured. ^*^ *P* < 0.05, ^**^ *P* < 0.01, and ^***^ *P* < 0.001 significantly different from the vehicle-treated group. Values represent the mean ± SEM of 10 mice.

KPFE (3, 10, or 30 mg/kg, s.c.) produced a dose-related antiedematogenic effect evidenced by the reduction in croton oil-induced mice ear edema by 50.8% (Δ = 2.9 ± 0.5 mg), 54.2% (Δ = 2.7 ± 0.7 mg), and 64.4% (Δ = 2.1 ± 0.5 mg), respectively, whereas dexamethasone (2 mg/kg) reduced the edema by 81.4% (Δ = 1.1 ± 0.3 mg) when compared with the vehicle treated group (Δ = 5.9 ± 1.0 mg), with ID_50_ 4.3 mg/kg (Figure 3(a)).

In another experimental set, pretreatment with the main flavonoid KPFV (0.3, 1.0, or 3.0 mg/kg, s.c.) also produced a dose-related antiedematogenic effect in the croton oil-induced mice ear edema by 38.2% (Δ = 4.2 ± 0.4 mg), 54.4% (Δ = 3.1 ± 0.4 mg), and 70.6% (Δ = 2.0 ± 0.4 mg), respectively, whereas the treatment with dexamethasone (2 mg/kg) reduced the ear edema by 85.3% (Δ = 1.0 ± 0.4 mg) when compared with the vehicle group (Δ = 6.8 ± 0.6 mg), with ID_50_ 0.76 mg/kg (Figure 3(b)).

**Figure 4:**
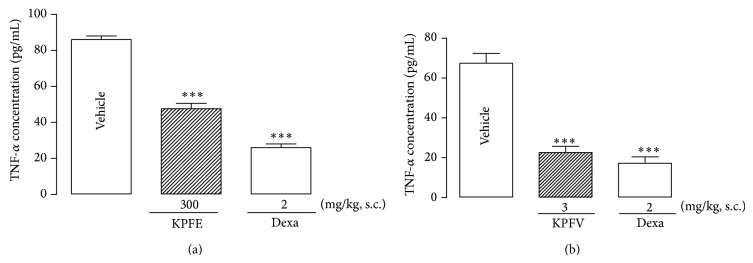
Effect of KPFE (a) and KPFV (b) on TNF-*α* concentration in the pleural exudates. KPFE, KPFV, or dexamethasone (Dexa) was subcutaneously administrated 30 min before carrageenan injection (1%, 0.1 mL/10 g). The pleural exudates were collected 4 h after the carrageenan injection. TNF-*α* concentration was determined by ELISA. ^***^ *P* < 0.001 significantly different from the vehicle-treated group. Values represent means ± SEM of the TNF-*α* concentration in the pleural exudates (pg/mL) of 8 mice per group.

After intrapleural injection of carrageenan, the pretreatment with KPFE (300 mg/kg, s.c.) or dexamethasone (2 mg/kg, s.c.) reduced the TNF-*α* concentration in pleural exudates (*P* < 0.001) by 44.7% (47.6 ± 0.3 pg/mL) and 69.8% (26.0 ± 2.0 pg/mL), respectively, when compared to the vehicle group (86.0 ± 2.0 pg/mL) (Figure 4(a)).

In an additional experiment, the flavonoid KPFV (3.0 mg/kg, s.c.) decreased the TNF-*α* concentration in pleural exudates by 66.6% (22.6 ± 3.1 pg/mL) when compared to the vehicle group (67.5 ± 4.9 pg/mL). As expected, dexamethasone (2 mg/kg, s.c.) also reduced the TNF-*α* concentration by 74.5% (17.2 ± 3.2 pg/mL) (Figure 4(b)).

The flavonoid KPFV inhibited both COX-1 and COX-2* in vitro* activities (Table 1), and the IC_50_ calculated for COX-1 inhibition was 3.8 × 10^−5^ M (22.1 *μ*g/mL). The maximum COX-2 inhibition induced by KPFV was 43.5% (50 *μ*g/mL); therefore the IC_50_ for COX-2 inhibition was >8.4 × 10^−5^ M. The selectivity index (SI; COX-1_IC_50__/COX-2_IC_50__) was <0.44. The positive control indomethacin also inhibited both COX-1 and COX-2 activities (IC_50_ for COX-1 and COX-2 was 5.9 and 31.2 *μ*g/mL, resp., and SI was 0.19).

## 4. Discussion

The anti-inflammatory potential of* K. pinnata* leaves was evaluated previously in different models [7–9]. Hema et al. [7] attributed the anti-inflammatory activity to the presence of sitosterol and aliphatic alcohols without testing any isolated compound. In the same way, there are also some reports that attribute this action to flavonoids [8, 9].

Our research group has been studying, via an interdisciplinary approach, the chemical composition of KP leaves taking into consideration their traditional use for healing wounds and other inflammatory processes. Our findings have demonstrated that KP flavonoids, isolated from the KP leaves extract, possess significant activity in leishmaniasis, asthma, and antiallergenic models, through immunomodulating mechanisms [3, 6].

**Figure 5:**
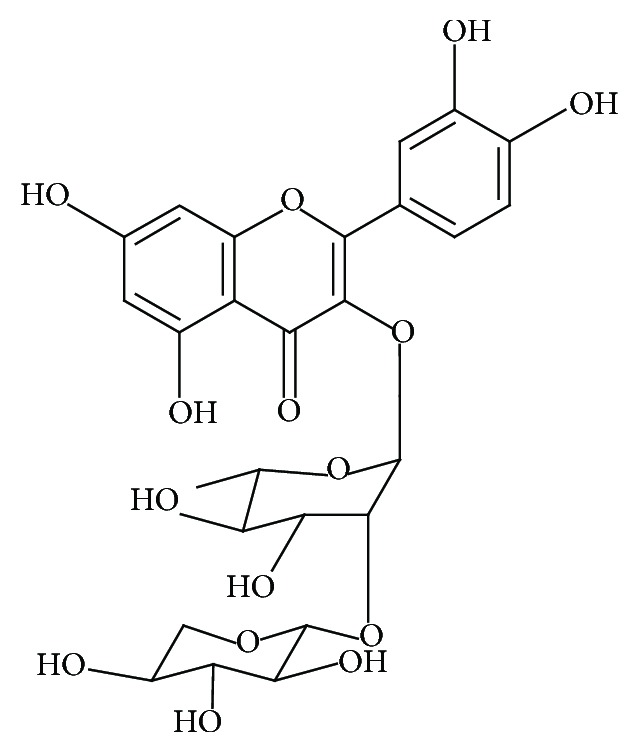
Structure of the main flavonoid from KP flowers: quercetin 3-*O*-*α*-L-arabinopyranosyl (1 → 2) *α*-L-rhamnopyranoside (KPFV).

Although the flowers present a higher content and variety of flavonoids, there are few studies of the isolation and characterization of bioactive molecules from the KP flowers or their pharmacological potential. Our previous study on the chemical composition of KP flowers led to the isolation of four flavonol glycosides, as well as a flavonol aglycone. Quercetin 3-*O*-*α*-L-arabinopyranosyl (1 → 2) *α*-L-rhamnopyranoside (KPFV) (Figure 5) proved to be the most abundant flavonoid in KP flowers (1.87% wt/wt) [10] and therefore is the aim of the present study. The other flavonoids isolated by our group were quercetin 3-*O*-*β*-glucuronopyranoside (miquelianin, 0.79% wt/wt), quercetin 3-*O*-*β*-glucopyranoside (isoquercitrin, 0.21% wt/wt), quercetin 3-*O*-*α*-L-rhamnopyranoside (quercitrin, 0.25% wt/wt), and quercetin (0.12% wt/wt). These flavonoids were considered to be the minority compounds [10].

The aglycone quercetin present in the chemical structure of KPFV proved to be an anti-inflammatory and immunosuppression agent [20, 21]. This flavonol has a well-known immunomodulatory effect through the regulation of inflammatory mediators, such as inhibiting cytokine and inducible nitric oxide synthase expression via inhibition of the NF-*κ* *β* pathway [15, 22]. Moreover, quercetin has been reported as having anti-inflammatory properties in experimental murine allergic asthma [23]. A study using human peripheral blood mononuclear cells showed the quercetin role in modulating the TNF-*α* production and gene expression, and this effect is linked to the modulation of the NF-*κ* *β*1 and I*κ* *β* pathways [24]. This flavonoid also inhibits PGE_2_ and nitric oxide production in IFN-*γ* and lipopolysaccharide-stimulated RAW 264.7 cells [25].

The content of the diglycosyl flavonoid KPFV in KP flowers is more than 2 times greater than the content of miquelianin. When comparing with isoquercitrin and quercitrin, the content of KPFV is 9 and 7 times higher, respectively. Recently we have shown that KPFV impairs T-cell proliferation (IC_50_ 38.8 *μ*g/mL) and it was able to inhibit the production of IL-2 and IL-4 cytokines [10]. The use of this flavonoid for antiallergenic purpose was the basis of a Japanese patent [26].

In present study the aqueous extract from* K. pinnata* flowers (KPFE) produced a dose-related inhibition of acetic acid-induced writhing indicating the antinociceptive activity. The main flavonoid (KPFV) also produced antinociceptive activity in the acetic acid-induced writhing model using doses up to 30 times lower than KPFE, suggesting the involvement of flavonoids, especially KPFV, in the antinociceptive effect of the flower extracts. The nociception induced by this assay is mediated by cyclooxygenase, such as PGE_2_, PGF_2*α*_, and PGI_2_, and lipoxygenase products, such as LTB_4_ [27, 28], which can explain its sensitivity by nonsteroidal anti-inflammatory drugs (NSAIDs) such as indomethacin [29].

Later, the anti-inflammatory effect of KPFE and the enriched-flavonoid fractions (EtOAcF and BuOHF) was evidenced by the reduction in the total leukocyte migration to the pleural cavity induced by carrageenan. This result could be explained, at least partially, by the high content of KPFV flavonoid in the EtOAcF (22.9%) and BuOHF (39.5%) fractions. Moreover, KPFV exhibited the same effect at doses up to 100 times lower than KPFE, demonstrating that KPFV is also involved in the anti-inflammatory effect of the flower extracts. Furthermore, carrageenan-induced leukocyte migration is dependent on the synthesis/release of the chemoattractants mediators leukotrienes such as LTB4 [30], cytokines IL-1 and TNF-*α* [31], and chemokines [32]. Pretreatment with KPFE or its main flavonoid KPFV reduced the TNF-*α* concentration in pleural exudates, suggesting that they produce an anti-inflammatory effect, at least in part, by TNF-*α* inhibition.

The anti-inflammatory activities of KPFE and its main flavonoid were also evidenced by the croton oil-induced ear edema test. Topical application of croton oil induces an acute inflammatory response mainly characterized by fluid accumulation and edema formation. The edema formation is initially mediated by histamine and serotonin and later by the release of prostaglandins [28]. 12-*O*-Tetradecanoylphorbol-13-acetate, a phorbol ester present in croton oil, has been reported to stimulate phospholipid-dependent protein kinase C and the overexpression of inducible nitric oxide synthase and cyclooxygenase-2 [33]. In this inflammatory model the dose-related inhibition of KPFE was detected at doses up to 100 times smaller (3 to 30 mg/kg) than used in leukocyte migration. Similarly, KPFV produced a reduction of edema formation and its effectiveness was obtained using lower doses when compared to KPFE and was active from 0.3 mg/kg. These findings are very promising considering that, recently, the anti-inflammatory activity of the leaf extract from KP was attributed to a novel steroid derivative, when Afzal et al. [34], using the model of carrageenan-induced rat paw edema and oral administration, showed that the extract (400 mg/kg) and the steroid (300 mg/kg) reduced the inflammation by 87% and 84%, respectively. These authors [34] needed to administer a high dose of the steroid compound from the leaf extract to obtain approximately the same effect as our results using the KPFV flavonoid. Furthermore, KPFE and KPFV produce antiedematogenic effect at lower doses than those required to produce antinociceptive effects, suggesting that they are more effective to produce the antiedematogenic and anti-inflammatory effects than the antinociceptive effect, as observed to other extracts or isolated compounds [35–37].

Prostaglandins play an important role in the setting of the cardinal signs of inflammation, pain, heat, redness, edema, and loss of function. The biosynthesis of PGE_2_, the main inflammatory prostaglandin, involves three key enzymes, phospholipase A_2_ (PLA_2_), cyclooxygenase (COX), and PGE synthase (PGES) [38]. Some flavonoids may reduce PGE_2_ synthesis by inhibiting the activity of these enzymes or by inhibiting the expression of the inflammatory-induced enzymes, COX-2, or microsomal PGES-1 [13, 39, 40]. Our results demonstrated that KPFV, as well as the positive control indomethacin, inhibited the activity of both COX-1 and COX-2. Its selectivity index (SI; COX-1_IC_50__/COX-2_IC_50__) was <0.44, indicating that KPFV has a slightly preferential inhibition to COX-1. These results indicate that KPFE and its main flavonoid also produce an anti-inflammatory effect by prostaglandins synthesis inhibition through COX inhibition. However, the involvement of other mechanisms of prostaglandins synthesis inhibition remains to be evaluated.

It is worth mentioning that quercetin 3-*O*-*α*-L-arabinopyranosyl (1 → 2) *α*-L-rhamnopyranoside (KPFV) is the most abundant flavonoid in both flowers (1.87% w/w) and leaves (2.26% w/w), as reported before [10]. Although this study has focused on the flowers of KP, it seems plausible that these results obtained with KPFV can be extended to the leaves. Thus, the presence of this flavonoid in the leaves must explain at least partially the popular use of the plant in inflammatory disorders.

## 5. Conclusions

For the first time the antinociceptive, antiedematogenic, and anti-inflammatory effects of* K. pinnata* flowers and its main flavonoid were described. These effects involve COX-1/COX-2 and TNF-*α* synthesis/release inhibition. The demonstration of the antinociceptive, antiedematogenic, and anti-inflammatory activities of the main flavonoid present in flowers and leaves [3, 10] of this species represents a major breakthrough in the pharmacological knowledge of a medicinal plant widely used in inflammatory processes.

## Supplementary Material

HPLC chromatograms of *Kalanchoe pinnata* flowers aqueous extract, organic fractions (EtOAcF and BuOHF), and isolated flavonoid [quercetin 3-O-*α*-L-arabinopyranosyl (1→2) *α*-L-rhamnopyranoside] (KPFV) are available on Supplementary Material. HPLC analyses were performed on a Shimadzu SPD-M10A VP instrument, using an RP-18 reverse-phase column (5 *μ*m, 250 mm x 4 mm, Lichro-CART/Lichrospher 100; Merck). The main flavonoid in *K. pinnata* is abundant in both fractions. ^1^H and ^13^ spectra of the flavonoid, recorded on a Varian MR-500 NMR spectrometer (^1^H: 500 MHz; ^13^C: 125 MHz), are also available.

## Figures and Tables

**Table 1:** Effect of flavonoid KPFV on COX-1 and COX-2 activities.

KPFV concentration (*µ*g/mL)	Inhibition (%)	
	COX-1	COX-2
3.125	44.2	18.5
6.25	46.1	20.4
12.5	49.0	26.9
25.0	50.0	38.9
50.0	ND	43.5
