# Supplementary material for: Mechanisms Underlying the Antinociceptive, Antiedematogenic, and Anti-Inflammatory Activity of the Main Flavonoid from Kalanchoe pinnata
Source: Evid Based Complement Alternat Med. 2014 Dec 11;2014:429256. doi: 10.1155/2014/429256 (PMC4279175; doi:10.1155/2014/429256)
Supplement: Supplementary file 1 — HPLC chromatograms of Kalanchoe pinnata flowers aqueous extract, organic fractions (EtOAcF and BuOHF), and isolated flavonoid [quercetin 3-O-α-L-arabinopyranosyl (1→2) α-L-rhamnopyranoside] (KPFV) are available on Supplementary Material. HPLC analyses were performed on a Shimadzu SPD-M10A VP instrument, using an RP-18 reverse-phase column (5 μm, 250 mm x 4 mm, Lichro-CART/Lichrospher 100; Merck). The main flavonoid in K. pinnata is abundant in both fractions. 1H and 13 spectra of the flavonoid, recorded on a Varian MR-500 NMR spectrometer (1H: 500 MHz; 13C: 125 MHz), are also available. [file 429256.f1.pdf]

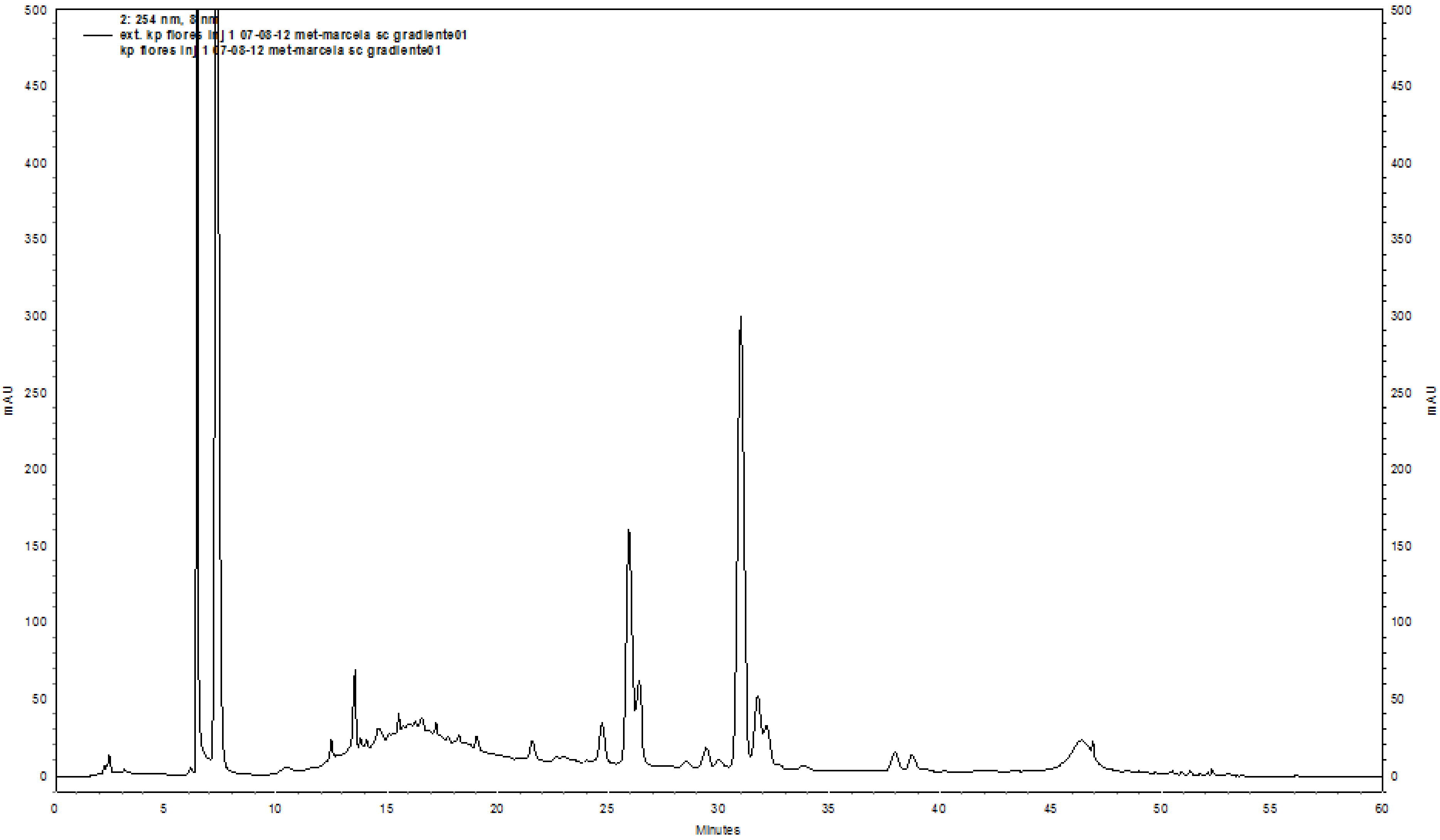

Fig. 1 HPLC chromatogram (254 nm) of aqueous extract from *Kalanchoe pinnata* flowers (sample: 10 mg/ml, volume injected: 20 μl, RP-18, H_2O- CH_3CN gradient).

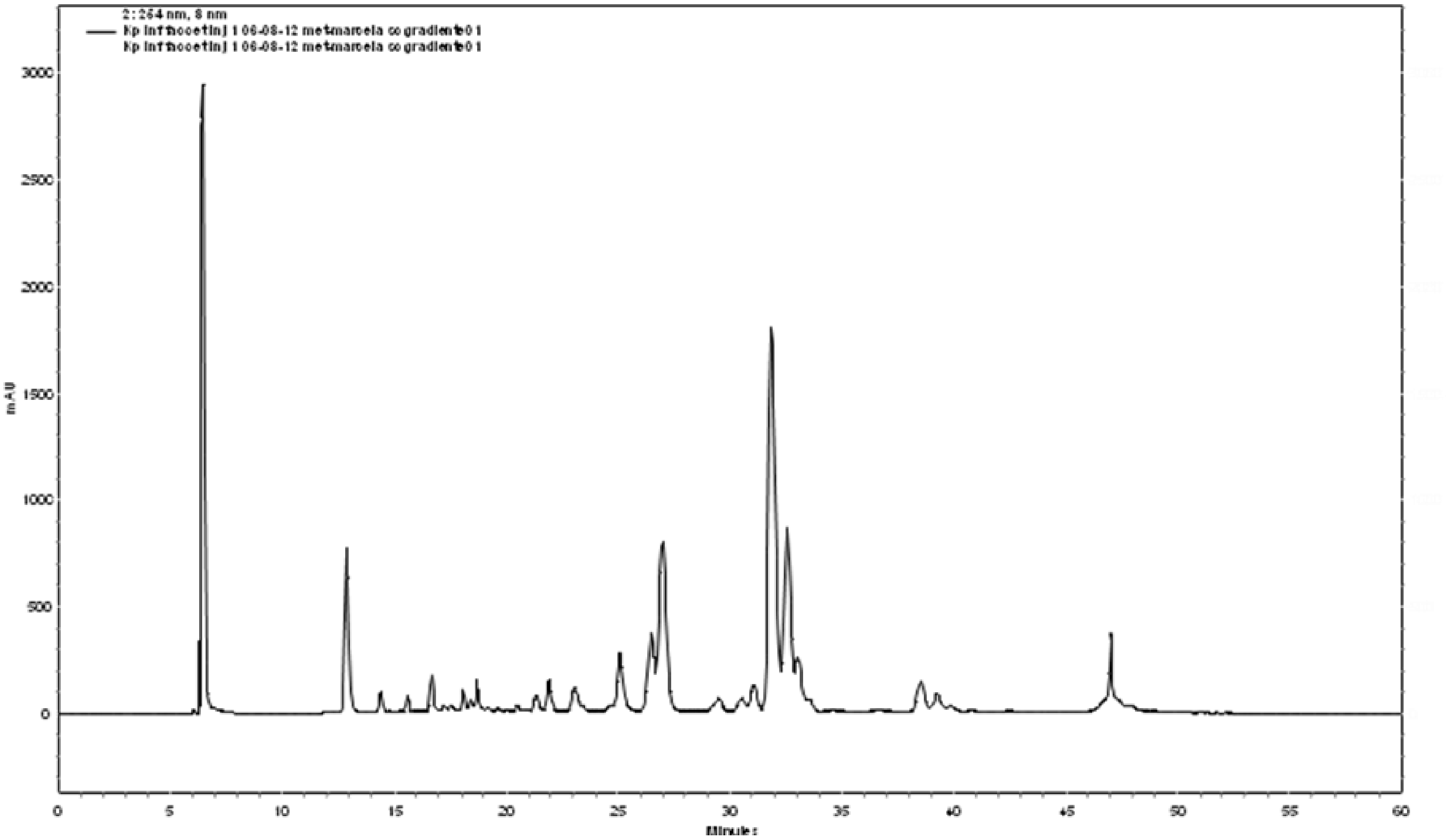

Fig. 2 HPLC chromatogram (254 nm) of ethyl acetate fraction (EtOAcF) from *Kalanchoe pinnata* flowers aqueous extract (sample: 10 mg/ml, volume injected: 20 μl, RP-18, H_2O- CH_3CN gradient).

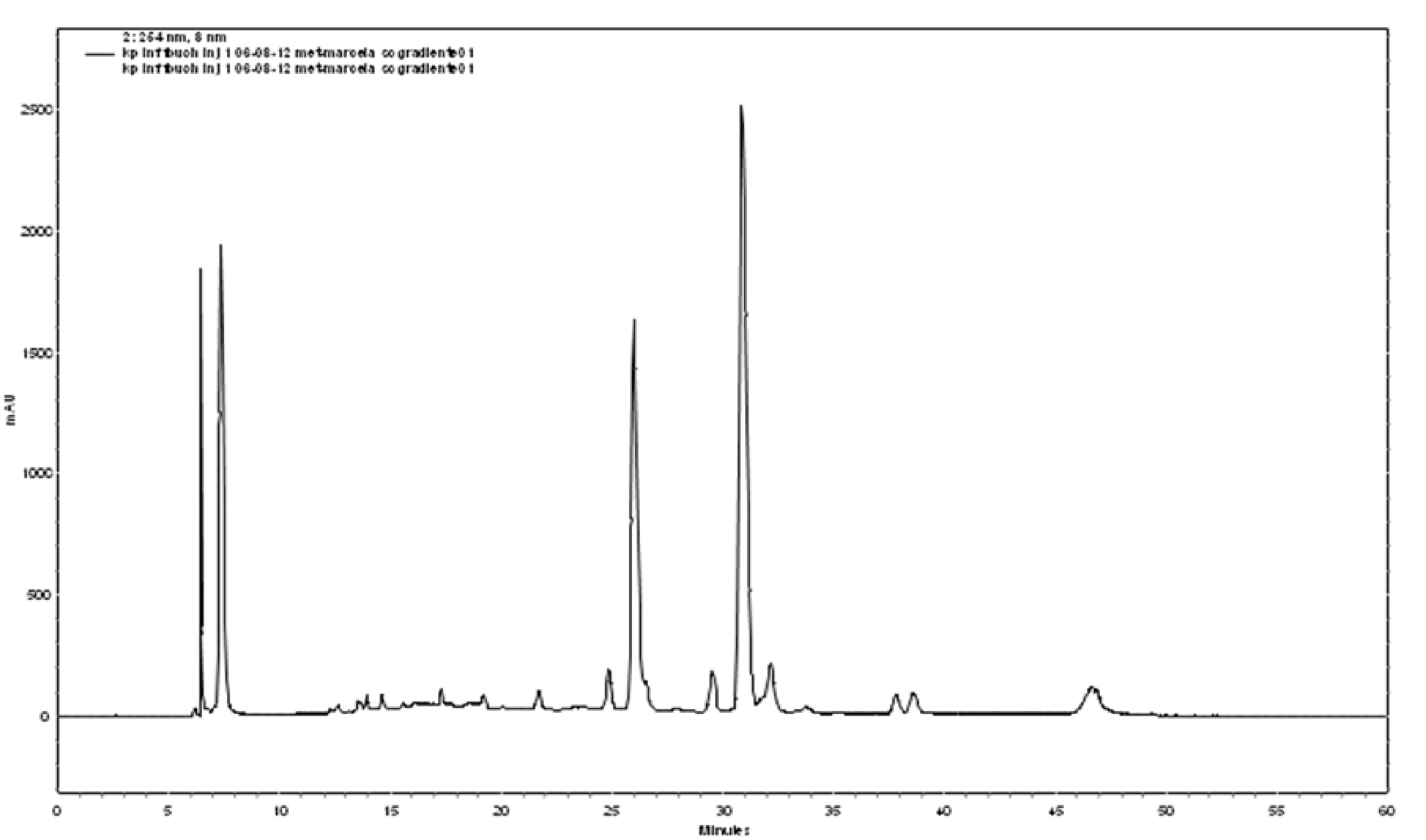

Fig. 3 HPLC chromatogram (254 nm) of butanolic fraction (BuOHF) from *Kalanchoe pinnata* flowers aqueous extract (sample: 10 mg/ml, volume injected: 20 μl, RP-18, H_2O- CH_3CN gradient).

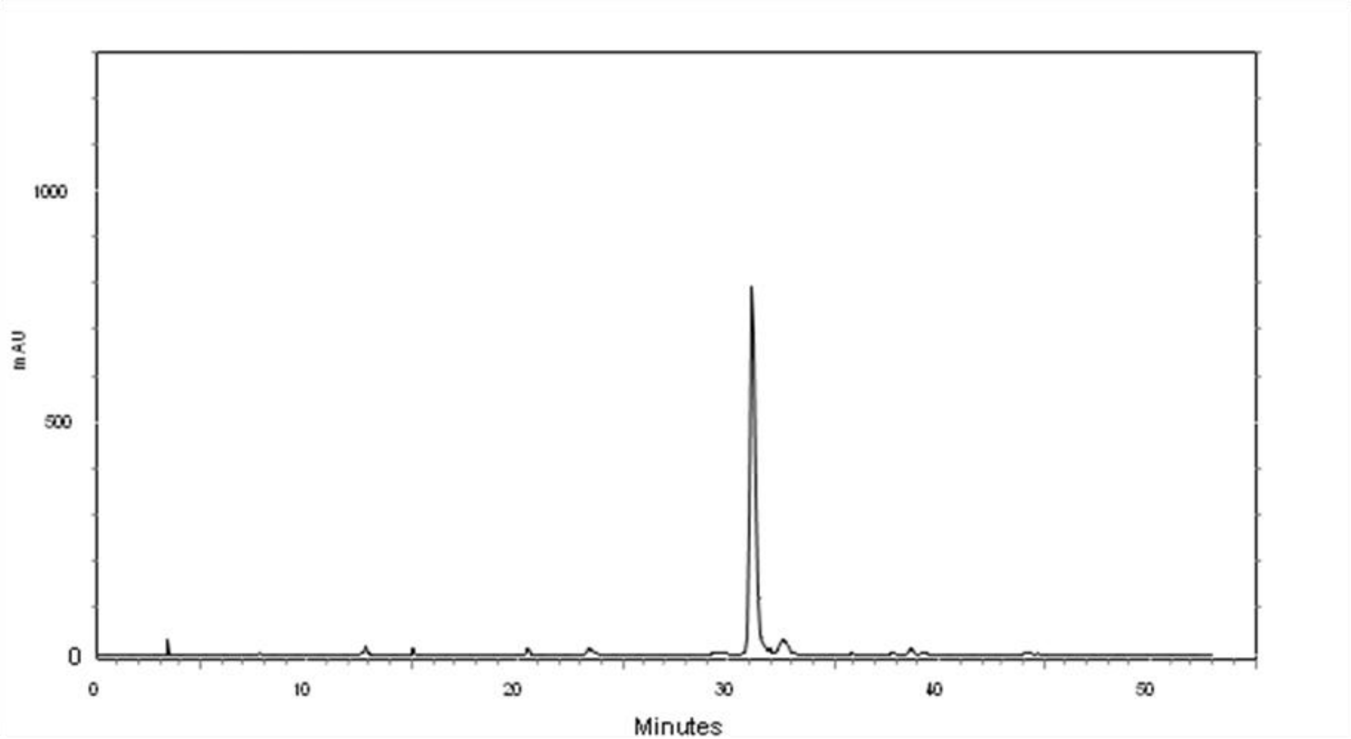

Fig. 4 HPLC chromatogram (254 nm) of the isolated flavonoid [quercetin 3-*O*-α-L-arabinopyranosyl (1→2) α-L-rhamnopyranoside] (KPFV) (Retention time: 31.2 minutes, sample: 1.0 mg/ml, volume injected: 20 μl, RP-18, H_2O- CH_3CN gradient).

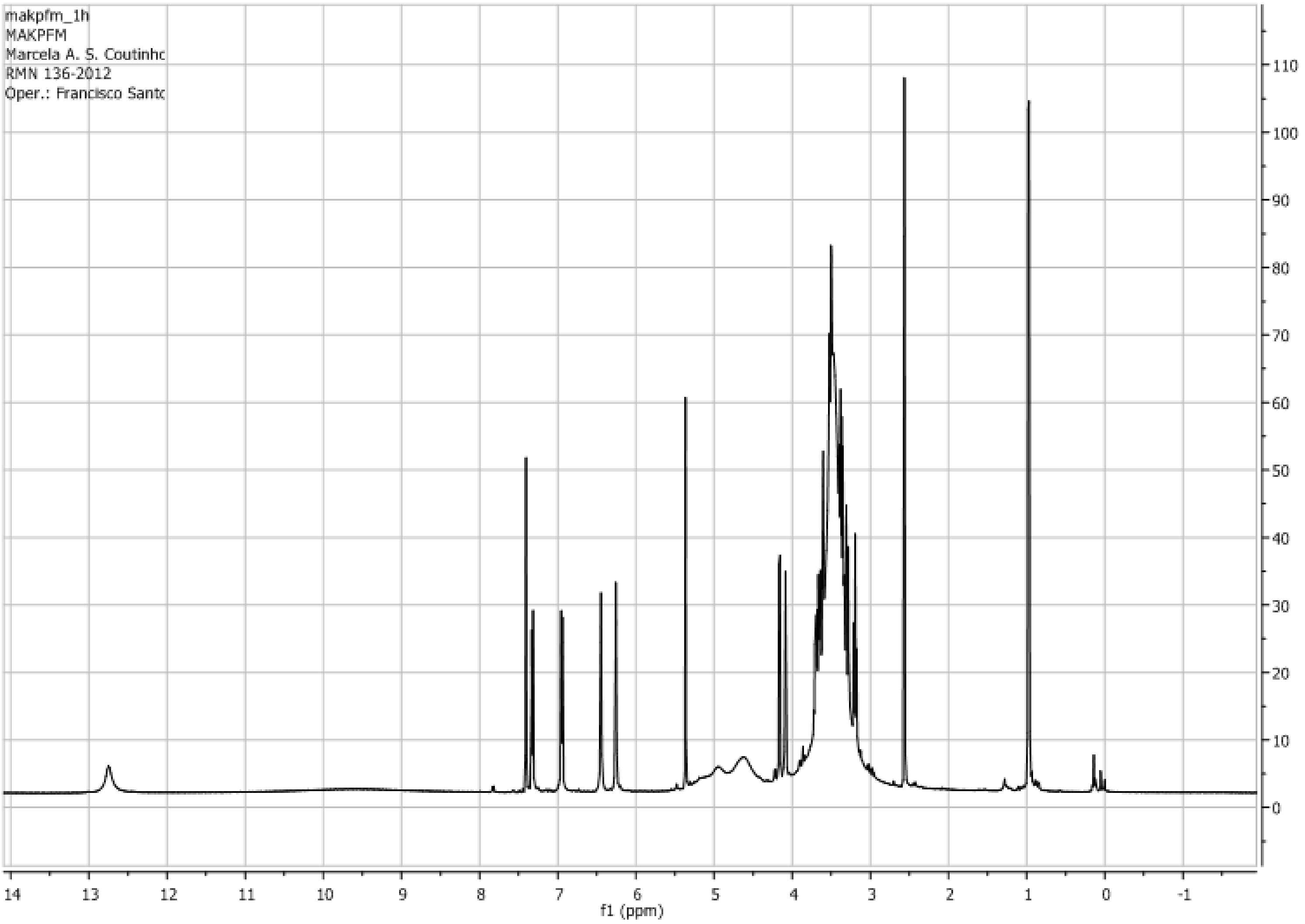

Fig. 5 ^{1}H NMR spectra (500 MHz; DMSO-d_6) of the isolated flavonoid [quercetin 3-*O*-α-L-arabinopyranosyl (1→2) α-L-rhamnopyranoside] (KPFV). Parameters: NS 16, SWH 8012 Hz, TD 32768, TMS, 25ºC.

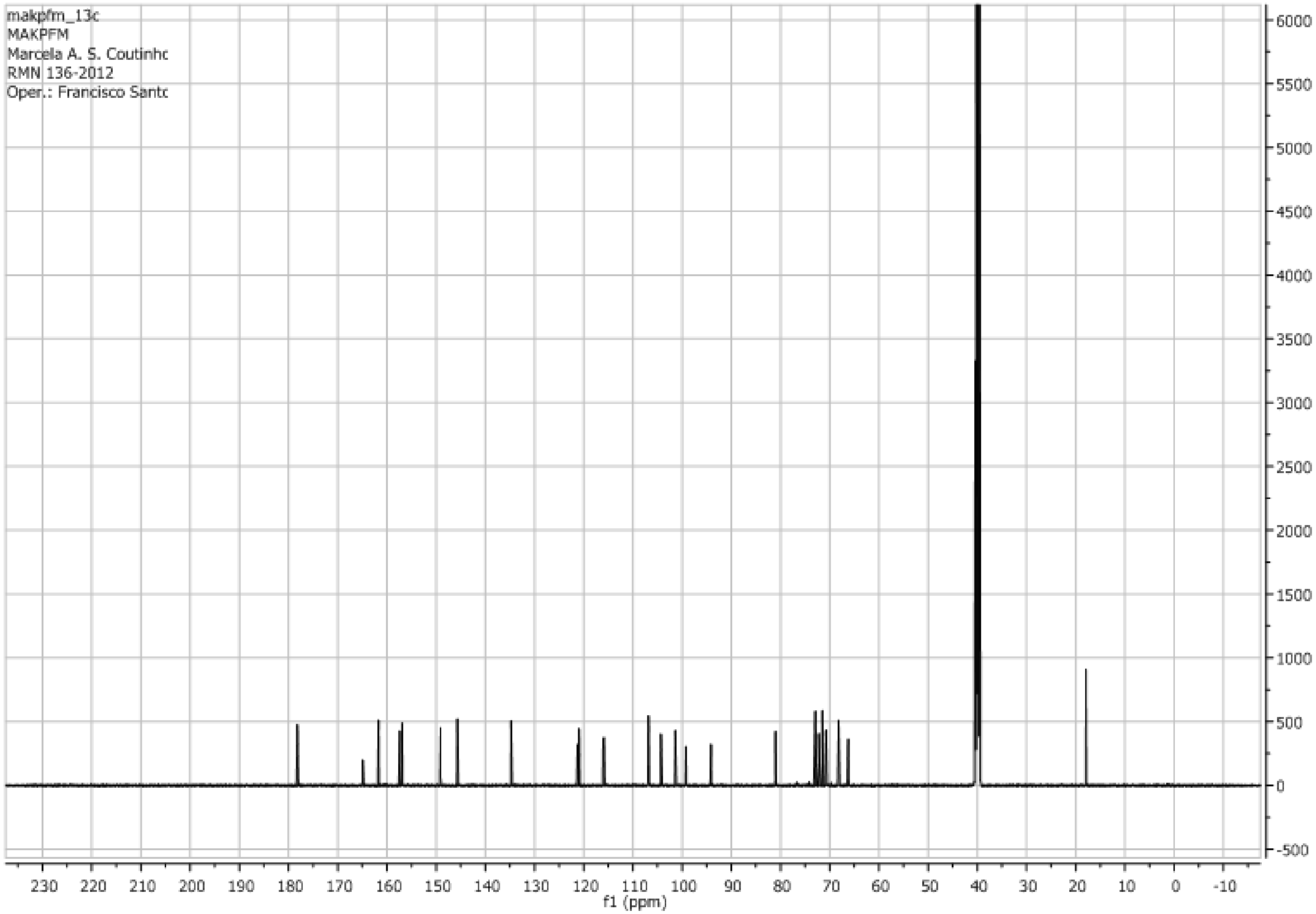

Fig. 6 ^{13}C NMR spectra (125 MHz; DMSO-d_6) of the isolated flavonoid [quercetin 3-*O*-α-L-arabinopyranosyl (1→2) α-L-rhamnopyranoside] (KPFV). Parameters: NS 98432, SWH 32051 Hz, TD 65536, TMS, 25ºC.
